# On Effusions into the Cavity of the Thorax, and a New Method of Evacuating Them

**Published:** 1841-07

**Authors:** 


*On Effusions into the Cavity of the Thorax, and a new method of evacuating them.* By M. Reybard.—The new method proposed by M. Reybard for the evacuation of fluids effused into the cavity of the thorax is, "to leave the opening which may be made in the thorax free during each act of expiration, but to close it during each inspiration," and thus prevent the ingress of air into the thoracic cavity, to which he attributes the numerous failures of the operation for empyema, &c. For this purpose he introduces into the wound a tube or canula, to the extremity of which is attached a portion of cat's intestine, softened by immersion in warm water, about three inches long and open at both ends. During expiration, this instrument gives free passage to any puss or fluid from the cavity of the thorax; but during inspiration the sides of the gut collapse, and, acting as a valve, completely prevent the air from entering the thorax.

Having performed several experiments with his valved canula on animals, and acquired a conviction of its utility, the author employed it on several occasions of empyema and hydrothorax, four of which he describes at considerable length. In one case, the patient was cured on the fifteenth day; in another, on the thirty-fifth day; in a third, it required four months of constant care before the wound in the thorax was completely healed.—*Provincial Medical and Surgical Journal. Ibid.*

